# Supplementary material for: Evaluating the frequency and the impact of pharmacogenetic alleles in an ancestrally diverse Biobank population
Source: J Transl Med. 2022 Nov 28;20:550. doi: 10.1186/s12967-022-03745-5 (PMC9703665; doi:10.1186/s12967-022-03745-5)
Supplement: Supplementary file 1 — Additional file 1: Figure S1. Demographic distribution on Penn Medicine EHR on left and Penn Medicine Biobank on the right. Table S1. Drugs queried for the study. Table S2. Numbers of samples with ambiguous and uncalled genotypes and phenotypes from PharmCAT. Table S3. CYP2D6 phenotype counts from PharmCAT research mode (without CNV and structural variants). Table S4. CYP2D6 activity score counts from PharmCAT research mode (without CNV and structural variants). [file 12967_2022_3745_MOESM1_ESM.docx]

**Additional Figure 1:** Demographic distribution on Penn Medicine EHR on left and Penn Medicine Biobank on the right.


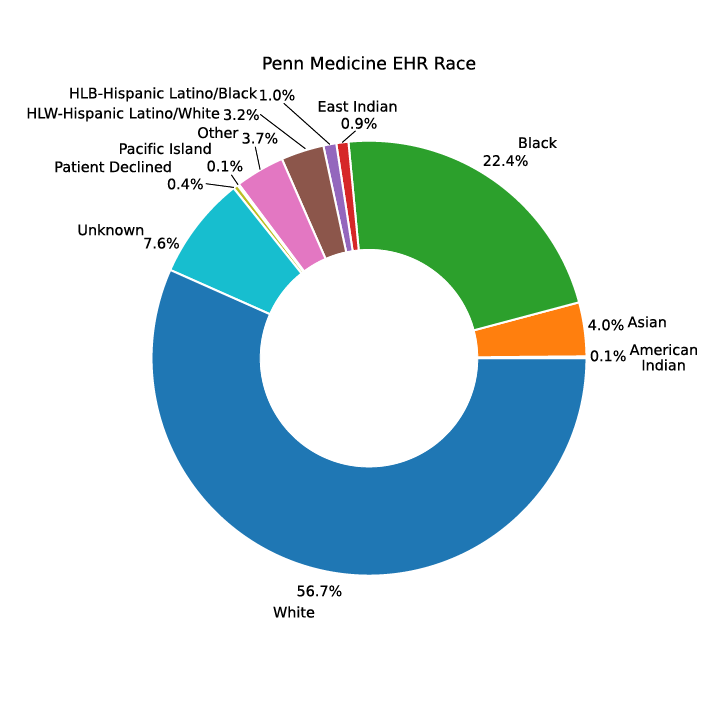

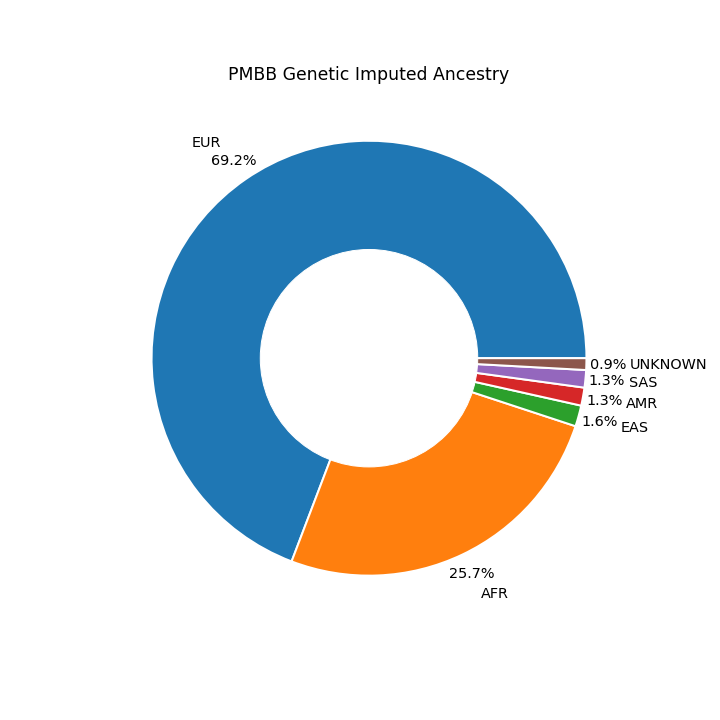

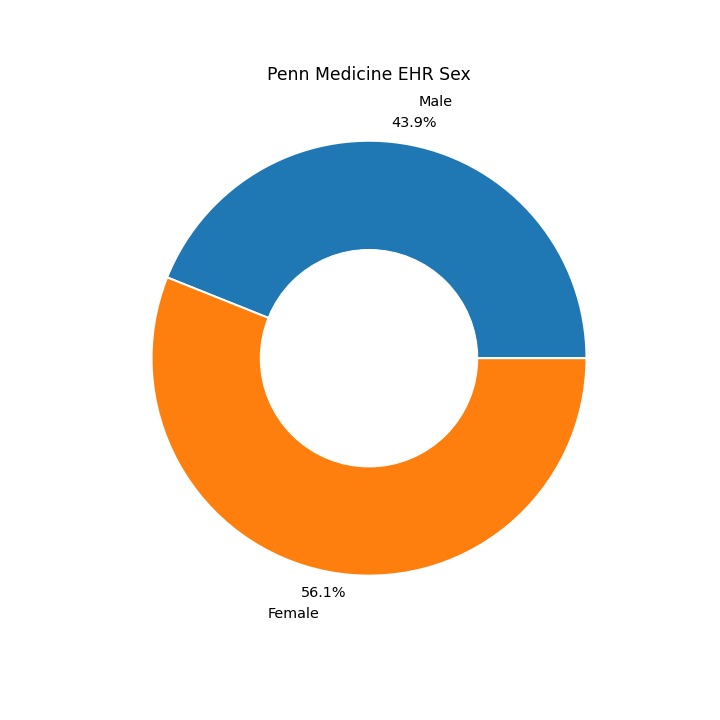

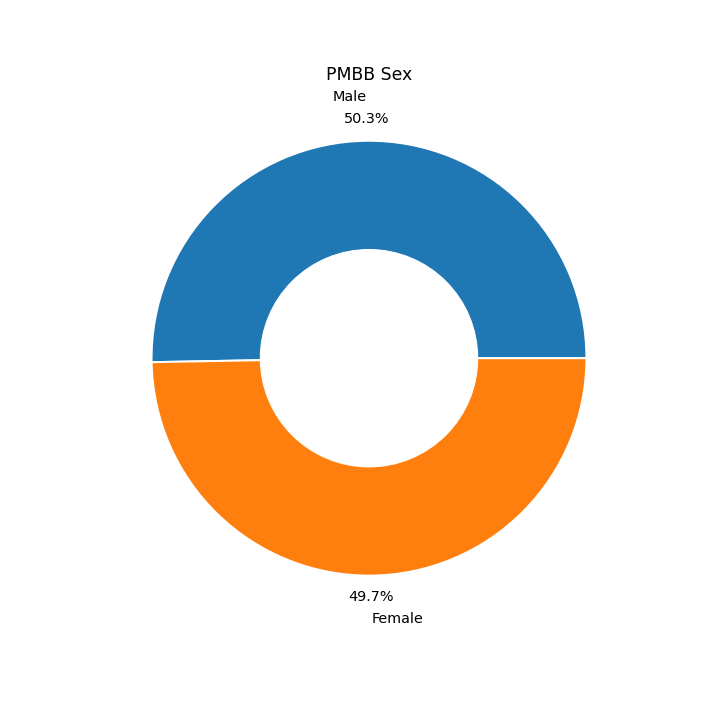


**Additional Table 1**: Drugs queried for the study

See *cpic_actionable.xlsx*

| **Medication** | **Indication** | **CPIC classification** |
| --- | --- | --- |
| Abacavir | HIV | A |
| Allopurinol | Gout | A |
| Amitriptyline | Depression | A |
| Atazanavir | HIV | A |
| Atomoxetine | ADHD | A |
| Azathioprine | Immunosuppression | A |
| Capecitabine | Cancer | A |
| Carbamazepine | Seizures | A |
| Celecoxib | Pain | A |
| Citalopram | Depression | A |
| Clomipramine | Depression | B |
| Clopidogrel | Myocardial infarction/stroke | A |
| Codeine | Pain | A |
| Desipramine | Depression | B |
| Dexlansoprazole | Acid reflux | B |
| Doxepin | Depression | B |
| Efavirenz | HIV | A |
| Escitalopram | Depression | A |
| Fluorouracil | Cancer | A |
| Flurbiprofen | Pain | A |
| Fluvoxamine | Depression | B |
| Fosphenytoin | Seizures | A |
| Hydrocodone | Pain | B |
| Ibuprofen | Pain | A |
| Imipramine | Depression | B |
| Ivacaftor | Cystic Fibrosis | A |
| Lansoprazole | Acid reflux | A |
| Meloxicam | Pain | A |
| Mercaptopurine | Immunosuppression | A |
| Nortriptyline | Depression | A |
| Omeprazole | Acid reflux | A |
| Ondansetron | Nausea/vomiting | A |
| Oxcarbazepine | Seizures | A |
| Pantoprazole | Acid reflux | A |
| Paroxetine | Depression | A |
| Peginterferon alfa-2a | Hepatitis C | A |
| Peginterferon alfa-2b | Hepatitis C | A |
| Phenytoin | Seizures | A |
| Piroxicam | Pain | A |
| Rasburicase | Hyperuricemia | A |
| Sertraline | Depression | B |
| Simvastatin | Hypercholesterolemia | A |
| Succinylcholine | Paralytic |  |
| Tacrolimus | Immunosuppression | A |
| Tamoxifen | Cancer | A |
| Thioguanine | Cancer | A |
| Tramadol | Pain | A |
| Voriconazole | Fungal infection | A |
| Warfarin | Thromboembolic disorders | A |
|  |  |  |
|  |  |  |

**Additional Table 2**: Numbers of samples with ambiguous and uncalled genotypes and phenotypes from PharmCAT

| *Gene/SNP* | # Not Called* | Proportion Not Called | # Ambiguous Genotype** | # Ambiguous Phenotype** | Proportion Ambiguous Genotype | Proportion Ambiguous Phenotype |
| --- | --- | --- | --- | --- | --- | --- |
| *CACNA1S* | 7 | 0.0002 | 0 | 0 | 0 | 0 |
| *CFTR* | 33 | 0.0008 | 0 | 0 | 0 | 0 |
| *CYP2B6* | 707 | 0.0163 | 13221 | 9 | 0.3049 | 0.0002 |
| *CYP2C19* | 121 | 0.0028 | 226 | 0 | 0.0052 | 0 |
| *CYP2D6* | 13536 | 0.3122 | 5984 | 2701 | 0.138 | 0.0623 |
| *rs12777823* | 1701 | 0.0392 | 0 | 0 | 0 | 0 |
| *CYP2C9* | 82 | 0.0019 | 4799 | 4710 | 0.1107 | 0.1086 |
| *CYP3A5* | 6 | 0.0001 | 0 | 0 | 0 | 0 |
| *CYP4F2* | 2679 | 0.0618 | 0 | 0 | 0 | 0 |
| *DPYD* | 16 | 0.0004 | 0 | 0 | 0 | 0 |
| *IFNL3* | 2265 | 0.0522 | 0 | 0 | 0 | 0 |
| *NUDT15* | 1 | 0 | 0 | 0 | 0 | 0 |
| *RYR1* | 9 | 0.0002 | 0 | 0 | 0 | 0 |
| *TPMT* | 22 | 0.0005 | 1 | 0 | 0 | 0 |
| *UGT1A1* | 940 | 0.0217 | 50 | 17 | 0.0012 | 0.0004 |
| *VKORC1* | 70 | 0.0016 | 0 | 0 | 0 | 0 |
| *SLCO1B1* | 0 | 0 | 917 | 515 | 0.0211 | 0.0119 |

*PharmCAT may fail to call samples when variants are missing from the input samples, either due to lack of coverage from the genotyping method or due to filtering during quality control. Furthermore, it can fail to call when the variants cannot be matched to an exact genotype in the allele definitions.

**Ambiguous genotypes are samples for which PharmCAT returned two or more genotypes with the same score based on matching the variant input to the allele definitions. An allele can be defined by one or more variant(s) and a variant can be present in several alleles. In case of unphased data, there are instances for which a variant input from the VCF file might match more than one genotype given the overlap in allele definitions. If PGx relevant positions are missing in the input data, those positions are ignored in the allele definitions. If that covers all variants in an allele definition, the allele cannot be considered in the matching process. If it only affects part of the variants in an allele definition, the allele is included in the matching process based on the remaining positions. Given the above-described overlap in allele definitions, missing positions can lead to definitions not being distinct from each other and the PharmCAT matcher provides all possible combinations with the highest score. Subsequently the PharmCAT Phenotyper assigns each genotype a corresponding phenotype based on the information stored in the CPIC database. In cases with two or more genotypes (ambiguous genotypes) the corresponding phenotypes were compared and if the phenotypes differ the sample was labeled as ambiguous phenotype. The above table lists the numbers of samples with ambiguous geno- and phenotypes. These samples were excluded from the analyses presented in the manuscript.

**Additional Table 3**: *CYP2D6* phenotype counts from PharmCAT research mode (without CNV and structural variants)

| *CYP2D6* Phenotype | Count |
| --- | --- |
| Normal Metabolizer | 7327 |
| Intermediate Metabolizer | 6330 |
| Poor Metabolizer | 924 |
| Ambiguous | 2701 |
| Indeterminate | 12541 |
| No Result | 13536 |

**Additional Table 4:** *CYP2D6* activity score counts from PharmCAT research mode (without CNV and structural variants)

| Activity Score | Count |
| --- | --- |
| Unknown | 28899 |
| 2 | 4248 |
| 1.5 | 2265 |
| 1.25 | 814 |
| 1 | 4991 |
| 0.75 | 91 |
| 0.5 | 685 |
| 0.25 | 442 |
| 0 | 924 |
