# Supplementary material for: Evaluating the frequency and the impact of pharmacogenetic alleles in an ancestrally diverse Biobank population
Source: J Transl Med. 2022 Nov 28;20:550. doi: 10.1186/s12967-022-03745-5 (PMC9703665; doi:10.1186/s12967-022-03745-5)
Supplement: Supplementary file 2 — Additional file 2: Regeneron Genetics Center Banner Author List and Contribution Statements. [file 12967_2022_3745_MOESM2_ESM.docx]

Regeneron Genetics Center Banner Author List and Contribution Statements

**RGC Management and Leadership Team**

Goncalo Abecasis, D.Phil. , Aris Baras, M.D. , Michael Cantor, M.D. , Giovanni Coppola, M.D. , Andrew Deubler , Aris Economides, Ph.D. , Katia Karalis, Ph.D. , Luca A. Lotta, M.D., Ph.D. , John D. Overton, Ph.D. , Jeffrey G. Reid, Ph.D. , Katherine Siminovitch, M.D. , Alan Shuldiner, M.D.

**Sequencing and Lab Operations**

Christina Beechert , Caitlin Forsythe, M.S. , Erin D. Fuller , Zhenhua Gu, M.S. , Michael Lattari , Alexander Lopez, M.S. , John D. Overton, Ph.D. , Maria Sotiropoulos Padilla, M.S. , Manasi Pradhan, M.S. , Kia Manoochehri, B.S. , Thomas D. Schleicher, M.S. , Louis Widom , Sarah E. Wolf, M.S. , Ricardo H. Ulloa, B.S.

**Clinical Informatics**

Amelia Averitt, Ph.D. , Nilanjana Banerjee, Ph.D. , Michael Cantor, M.D. , Dadong Li, Ph.D. , Sameer Malhotra, M.D. , Deepika Sharma, MHI , Jeffrey Staples , Ph.D.

**Genome Informatics**

Xiaodong Bai, Ph.D. , Suganthi Balasubramanian, Ph.D. , Suying Bao, Ph.D. , Boris Boutkov, Ph.D. , Siying Chen, Ph.D. , Gisu Eom, B.S. , Lukas Habegger, Ph.D. , Alicia Hawes, B.S. , Shareef Khalid , Olga Krasheninina, M.S. , Rouel Lanche, B.S. , Adam J. Mansfield, B.A. , Evan K. Maxwell, Ph.D. , George Mitra, B.A. , Mona Nafde, M.S. , Sean O’Keeffe, Ph.D. , Max Orelus, B.B.A. , Razvan Panea, Ph.D. , Tommy Polanco, B.A. , Ayesha Rasool, M.S. , Jeffrey G. Reid, Ph.D. , William Salerno, Ph.D. , Jeffrey C. Staples, Ph.D. , Kathie Sun, Ph.D.

**Analytical Genomics and Data Science**

Goncalo Abecasis, D.Phil. , Joshua Backman, Ph.D. , Amy Damask, Ph.D. , Lee Dobbyn, Ph.D. , Manuel Allen Revez Ferreira, Ph.D. , Arkopravo Ghosh, M.S. , Christopher Gillies, Ph.D. , Lauren Gurski, B.S. , Eric Jorgenson, Ph.D. , Hyun Min Kang, Ph.D. , Michael Kessler, Ph.D. , Jack Kosmicki, Ph.D. , Alexander Li , Ph.D. , Nan Lin, Ph.D. , Daren Liu, M.S. , Adam Locke, Ph.D. , Jonathan Marchini, Ph.D. , Anthony Marcketta, M.S. , Joelle Mbatchou, Ph.D. , Arden Moscati, Ph.D. , Charles Paulding, Ph.D. , Carlo Sidore, Ph.D. , Eli Stahl, Ph.D. , Kyoko Watanabe, Ph.D. , Bin Ye, Ph.D. , Blair Zhang, Ph.D. , Andrey Ziyatdinov, Ph.D.

**Therapeutic Area Genetics**

Ariane Ayer, B.S. , Aysegul Guvenek, Ph.D. , George Hindy, Ph.D. , Giovanni Coppola, M.D. , Jan Freudenberg, M.D. , Jonas Bovijn M.D. , Katherine Siminovitch, M.D. , Kavita Praveen, Ph.D. , Luca A. Lotta, M.D. , Manav Kapoor, Ph.D. , Mary Haas, Ph.D. , Moeen Riaz , Ph.D. , Niek Verweij, Ph.D. , Olukayode Sosina, Ph.D. , Parsa Akbari, Ph.D. , Priyanka Nakka, Ph.D. , Sahar Gelfman, Ph.D. , Sujit Gokhale, B.E. , Tanima De, Ph.D. , Veera Rajagopal, Ph.D. , Alan Shuldiner, M.D. , Bin Ye, Ph.D. , Gannie Tzoneva, Ph.D. , Juan Rodriguez-Flores, Ph.D.

**RGC Biology**

Shek Man Chim, Ph.D. , Valerio Donato, Ph.D. , Aris Economides, Ph.D. , Daniel Fernandez, M.S. , Giusy Della Gatta, Ph.D. , Alessandro Di Gioia, Ph.D. , Kristen Howell, M.S. , Katia Karalis, Ph.D. , Lori Khrimian, Ph.D. , Minhee Kim, Ph.D. , Hector Martinez , Lawrence Miloscio, B.S. , Sheilyn Nunez, B.S. , Elias Pavlopoulos, Ph.D. , Trikaldarshi Persaud, B.S.

**Research Program Management & Strategic Initiatives**

Esteban Chen, M.S. , Marcus B. Jones, Ph.D. , Michelle G. LeBlanc, Ph.D. , Jason Mighty, Ph.D. , Lyndon J. Mitnaul, Ph.D. , Nirupama Nishtala, Ph.D. , Nadia Rana, Ph.D.
